# Clinical determinants of survival outcomes and acute toxicity after adoptive cellular therapy for solid tumors

**DOI:** 10.3389/fimmu.2026.1848516

**Published:** 2026-07-29

**Authors:** Jeong Uk Lim, Derrick Tao, Paul Nevins Selvadurai, Ecaterina Elena Dumbrava, Aung Naing, Stephane Champiat, Oriol Mirallas, Lei Kang, Hung Le, Apostolia Maria Tsimberidou, Jordi Rodon Ahnert, Sarina A. Piha-Paul, Siqing Fu, Timothy A. Yap, Tin-Yun Tang, Funda Meric-Bernstam, Ajay Sheshadri, David S. Hong

**Affiliations:** 1 Department of Investigational Cancer Therapeutics, The University of Texas MD Anderson Cancer Center, Houston, TX, United States; 2 Division of Pulmonary, Allergy and Critical Care Medicine, Department of Internal Medicine, Yeouido St. Mary’s Hospital, College of Medicine, The Catholic University of Korea, Seoul, Republic of Korea; 3 Melanoma & Skin Cancer Center, Division of Hematology/Oncology, Department of Medicine, University of California, San Francisco, San Francisco, CA, United States; 4 Department of Pulmonary Medicine, The University of Texas MD Anderson Cancer Center, Houston, TX, United States

**Keywords:** adoptive cell therapy (ACT), cytokine release syndrome, immune effector cell-associated neurotoxicity syndrome (ICANS), solid tumor, T cells

## Abstract

**Background:**

The efficacy of cellular therapies has been demonstrated in hematologic malignancies, and their use is expanding to solid tumors. Predictors of outcomes and toxicities remain limited in patients undergoing cellular therapies for solid tumors.

**Methods:**

Data on patients with solid tumors who received cellular therapies between January 2016 and July 2025 in the Department of Investigational Cancer Therapeutics (Phase I Clinical Trials Program) at The University of Texas MD Anderson Cancer Center were reviewed retrospectively using the institutional CHIMERA database platform.

**Results:**

Among 122 patients, increased baseline C-reactive protein (CRP) was associated with shorter overall survival (OS) (hazard ratio [HR] 1.009, 95% confidence interval (CI) 1.005–1.013; P < 0.001), and higher log-transformed cell dose was associated with longer progression-free survival (PFS) (HR 0.636, 95% CI 0.476–0.850; P = 0.002). Among patients with baseline pulmonary function testing, zDLCO was associated with OS (HR 0.794, 95% CI 0.674-0.936; P = 0.006) and PFS (HR 0.826, 95% CI 0.696-0.979; P = 0.028). Cytokine release syndrome (CRS) occurred in 70 participants (57.4%). In the multivariate analysis, lower baseline absolute neutrophil count was associated with CRS of any grade (odds ratio [OR] 0.594, 95% CI 0.376-0.939; P = 0.026) and with CRS grade 2 or higher (OR 0.470, 95% CI 0.252-0.877; P = 0.018). Immune effector cell-associated neurotoxicity syndrome developed in 11 patients (9.0%).

**Conclusions:**

Baseline parameters such as CRP and pulmonary function may help identify patients at higher risk of adverse outcomes and toxicity during cellular therapy for solid tumors, and warrant prospective evaluation.

## 1 Introduction

Cellular therapies, particularly chimeric antigen receptor T-cell (CAR-T) therapies, are important therapies for hematologic malignancies, such as acute lymphocytic leukemia, B-cell lymphoma and multiple myeloma (1–7), and the use of cellular therapies is now expanding to solid tumors (8). Although the efficacy of cellular therapies in solid tumors has been shown in early trials (9, 10), relatively little attention has been given to predictors of toxicity and long-term survival. In patients with hematologic cancers undergoing cellular therapies, tumor burden, prior lines of treatment, and cytokine levels are correlated with survival outcomes (11–14). Similar studies investigating predictors of outcomes in patients with solid tumors remain limited.

In particular, understanding the impact of comorbidities and baseline organ function may offer prognostic value. In hematopoietic cell transplantation (HCT), pre-HCT pulmonary function testing (PFT) predicts the risk for early mortality and respiratory failure (15). Similarly, a retrospective study showed that pre–CAR-T PFTs may predict toxicities in patients undergoing CAR-T therapy for lymphoma (16). However physiologic assessments such as PFTs are not routinely performed prior to cellular therapies for solid tumors. In solid tumor cellular therapies, there are no predictive models for severe toxicities such as cytokine release syndrome (CRS) and immune effector cell–associated neurotoxicity syndrome (ICANS), which can be life-threatening (17).

In the present study, we performed a retrospective analysis of participants with solid tumors receiving cellular therapies to identify predictors of efficacy, safety, and survival.

## 2 Methods

### 2.1 Patients and data source

Data on patients with solid tumors who received cellular therapies between January 2016 and July 2025 in the Department of Investigational Cancer Therapeutics at The University of Texas MD Anderson Cancer Center, a dedicated Phase I Clinical Trials Program, were reviewed retrospectively using the MD Anderson CHIMERA database platform. Among 125 patients who received cell therapy for solid tumors, three patients with documented bacteremia were excluded because sepsis could not be reliably distinguished from cytokine release syndrome. A total of 122 patients were included in the final analysis. All analyses were performed under protocol RCR05-0270, which was approved by the Institutional Review Board of MD Anderson Cancer Center.

### 2.2 Covariates

Collected covariates included demographic and clinical characteristics such as age, sex, race, smoking history, body mass index (BMI), and Eastern Cooperative Oncology Group (ECOG) performance status. Tumor-related variables included histology, presence of specific metastatic sites (brain, liver, lung or pleura), and overall metastatic burden, defined by the number of metastatic lesions. Treatment history was recorded, including prior exposure to cytotoxic chemotherapy, immunotherapy, targeted therapy, hormonal therapy, radiotherapy, and the total number of systemic therapy lines. Treatment-related variables included the type of infused product (T cell or NK cell), infused cell dose, and use of interleukin 2 agonist in addition to cell infusion. From the treatment records, the number of infused cells was obtained as an absolute value (×10^6^ cells) and adjusted for body weight (cells/kg). To account for skewed distribution, the total infusion count was transformed to a base-10 logarithmic scale (log_10_) prior to analysis.

Laboratory data were also acquired. Pre–cell infusion baseline data closest to the infusion day (Day –1 to –3) were collected for absolute neutrophil count (ANC), absolute lymphocyte count (ALC), absolute monocyte count (AMC), absolute eosinophil count (AEC), platelet count, and C-reactive protein (CRP).

### 2.3 Underlying comorbidities

Comorbidities were determined based on documented patient history, regular outpatient medications, and clinical evaluations, including specialist consultations when applicable. Single prescriptions, isolated diagnosis codes, or provisional impressions without corroborating evidence were not considered sufficient for classification as a significant comorbidity.

### 2.4 Pulmonary function testing

For patients who underwent PFTs within 12 months prior to cell infusion, we extracted percent-predicted values for forced expiratory volume in 1 second (FEV_1_%), forced vital capacity (FVC%), diffusing capacity for carbon monoxide (DLCO%), and corrected DLCO (cDLCO%) as well as z-scores (zFEV_1_, zFVC, and zDLCO) using Global Lung Function Initiative (GLI) reference equations (18–20). The z-scores take patient factors such as age, sex, height, and ethnicity into account. A z-score greater than −1.645 is considered within the normal range, values between −1.65 and −2.5 indicate mild impairment, between −2.5 and −4 represent moderate impairment, and those below −4 correspond to severe impairment (21). PFT prior to treatment was not mandatory and ordering this is up to the physician’s discretion. PFT was performed according to American Thoracic Society/European Respiratory Society (ATS/ERS) protocols (19, 21). In addition, a composite Lung Function Score (LFS) was derived from FEV_1_ and DLCO values. The LFS was calculated using FEV1 and cDLCO. Percent-predicted values for each parameter were converted into numeric scores as follows: >80% = 1, 70–79% = 2, 60–69% = 3, 50–59% = 4, 40–49% = 5, and <40% = 6. Subsequently, the numeric scores were summed up, and the combined values were sorted into following groups: LFS 0 (normal) = 2, LFS 1 (mild) = 3-5, LFS 2 (moderate)= 6- 9, LFS 3 (severe)= 10-12 (16, 22).

### 2.5 CRS and ICANS assessment

CRS and ICANS were monitored prospectively as part of institutional inpatient protocols following cell infusion. Assessment and grading of CRS and ICANS were performed based on the consensus criteria established by the American Society for Transplantation and Cellular Therapy (17). According to the protocol, patients were monitored with a focus on clinical parameters such as fever, hypotension, and hypoxia for CRS and its grade, while for ICANS, monitoring focused on the Immune Effector Cell–associated Encephalopathy (ICE) score, level of consciousness, presence of seizures, motor findings, and signs or symptoms indicative of increased intracranial pressure.

### 2.6 Statistical analysis

All analyses were conducted using SPSS version 22 (IBM Corp., Armonk, NY) and Stata version 18 (StataCorp, College Station, TX). Continuous variables were summarized as medians with ranges and compared using nonparametric tests. Categorical variables were summarized as frequencies with percentages and compared using chi-square or Fisher’s exact tests, as appropriate. Logistic regression models were used to evaluate associations between patient or disease characteristics and binary outcomes including CRS and ICANS. Cox proportional hazards regression models were fit for progression free survival (PFS) and overall survival (OS). The proportional hazards assumption was verified for each model using scaled Schoenfeld residuals. PFS was defined as the time from cell infusion (C1D1) to documented disease progression or last follow-up. OS was defined as the time from cell infusion to death from any cause or last follow-up. The data cutoff was August 30, 2025.

Covariates for the multivariable models were selected *a priori* on the basis of established clinical and biological relevance (23), rather than their significance in univariate testing. The number of covariates in each model was kept proportionate to the number of observed events to minimize overfitting. For the two endpoints, CRS of grade 3 or higher and ICANS, the number of events was insufficient for reliable multivariable modeling, and only univariate associations are reported.

Missing covariate values in the toxicity models were addressed using multiple imputation by chained equations, with estimates combined across the imputed datasets using Rubin’s rules; the survival models were analyzed using complete cases. All tests were two-sided, and a P value below 0.05 was considered statistically significant.

## 3 Results

### 3.1 Patient characteristics

A total of 122 patients were included (**Table 1**). The median age was 57 years (range, 20–84), and 74 patients (60.7%) were female. The majority were White (87.7%), and 61.3% were never-smokers. The most frequent tumor types were gynecologic cancers (18.0%), followed by sarcoma (17.2%), and gastrointestinal cancers (14.8%). Metastatic involvement was present in the lung or pleura in 61 patients (50.0%), liver in 36 (29.5%), and brain in 10 (8.2%). More than half of patients (63.1%) had received three or more prior systemic therapy lines. Cytotoxic chemotherapy was the most common prior treatment (88.5%), followed by targeted therapy (59.8%), and immunotherapy (49.2%). ECOG performance status was 1 in most patients (74.6%). The median BMI was 25.7 (range, 17.1–48.2). T-cell–based products were infused in 103 patients (84.4%), and NK-cell–based products in 19 (15.6%). Twenty-four patients (19.7%) received IL-2 agonist in addition to cell infusion.

**Table 1:** Patient characteristics.

Parameter	N (%)
Number	122
Demographics	
Age (median (range)) years	57 (20-84)
Sex	
Male	48 (39.3)
Female	74 (60.7)
Smoking history^a^	
Never smoker	73 (61.3)
Ever smoker	46 (38.7)
Race	
White	107 (87.7)
Black or African American	4 (3.3)
Asian	3 (2.5)
Hispanic or Latino	1 (0.8)
Other	7 (5.7)
Histology	
Breast	14 (11.5)
Gastrointestinal	18 (14.8)
Head and neck	16 (13.1)
Lung	3 (2.5)
Melanoma	13 (10.7)
Mesothelioma	11 (9.0)
Gynecological	22 (18.0)
Sarcoma	21 (17.2)
Prostate	4 (3.3)
Metastases	
0	19 (15.6)
1	43 (35.2)
2	37 (30.3)
3 or more	23 (18.9)
Brain metastases	10 (8.2)
Liver metastases	36 (29.5)
Lung or pleura metastases	61 (50.0)
Prior lines of treatment	
0	6 (4.9)
1	16 (13.1)
2	23 (18.9)
3 or more	77 (63.1)
Chemotherapy	104 (88.5)
Targeted therapy	73 (59.8)
Immunotherapy	60 (49.2)
Targeted and immunotherapy combination	5 (4.1)
Hormonal therapy	13 (10.7)
Other therapy	4 (3.3)
Prior radiotherapy	57 (46.7)
Prior radiotherapy to thorax	14 (11.5)
BMI (median (range))	25.7 (17.1-48.2)
ECOG	
0	29 (23.8)
1	91 (74.6)
2 or more	2 (1.6)
Cell therapy	
T-cell	103 (84.4)
NK cell	19 (15.6)
IL2 agonist addition to cell infusion	24 (19.7)
Underlying comorbidities	
Asthma	15 (12.3)
COPD	2 (1.6)
Prior pneumonia	4 (3.3)
Prior pneumonitis	1 (0.8)
Pleural effusion	23 (18.9)
Autoimmune disease	4 (3.3)
Cardiac disease	8 (6.6)
Baseline laboratory results at cell infusion	
Absolute neutrophil count (median (range))	1070 (0-4740)
Absolute lymphocyte count (median (range))	30 (0-1470)
Absolute monocyte count (median (range))	40 (0-800)
Absolute eosinophil count (median (range))	60 (0-930)
Platelet count	167 (43-386)
C-reactive protein^b^	13.8 (0.4-248.8)
Pulmonary function tests^c^	
FEV1 (%) (mean (SD))	88 (19)
FVC (%) (mean (SD))	88 (18)
DLCO (%) (mean (SD))	83 (19)
cDLCO (%) (mean (SD))	85 (19)
zFEV1 (median (range))	-0.6 (-4.1 - 2.4)
zFVC (median (range))	-0.5 (-3.8 - 1.9)
zDLCO (median (range))	-1.1 (-8.7 - 1.4)

aThree patients have missing data, and percentages were calculated among patients with available data.

b Five patients with missing data.

c Sixty-three patients underwent pulmonary functions tests. BMI, body mass index; COPD, chronic obstructive pulmonary disease; CRS, cytokine release syndrome; ECOG, Eastern Cooperative Oncology Group; FEV1, forced expiratory volume in 1 second; FVC, forced vital capacity; DLCO, diffusing capacity of the lung for carbon monoxide; cDLCO, hemoglobin-corrected diffusing capacity of the lung for carbon monoxide; zFEV1, z-score of forced expiratory volume in 1 second; zFVC, z-score of forced vital capacity; zDLCO, z-score of diffusing capacity of the lung for carbon monoxide; PFT, pulmonary function test; CRP, C-reactive protein; IL2, interleukin-2; NK cell, natural killer cell.

### 3.2 Comparison between patients with and without available PFT data

A total of 63 patients had available PFT results, while 59 patients did not. Patients with PFT had a significantly lower proportion of those with three or more prior lines of treatment (*P* = 0.037) and poor ECOG performance status (*P* = 0.031), but a higher proportion who received IL-2 agonist addition to cell infusion (*P* = 0.011). No significant differences were observed in median PFS or OS between the two groups, although patients in the PFT group showed a significantly higher incidence of CRS of any grade compared to the non-PFT group (69.8% vs 44.1%, P = 0.004) (**Supplementary Table 1**).

### 3.3 OS

**Figure 1:**
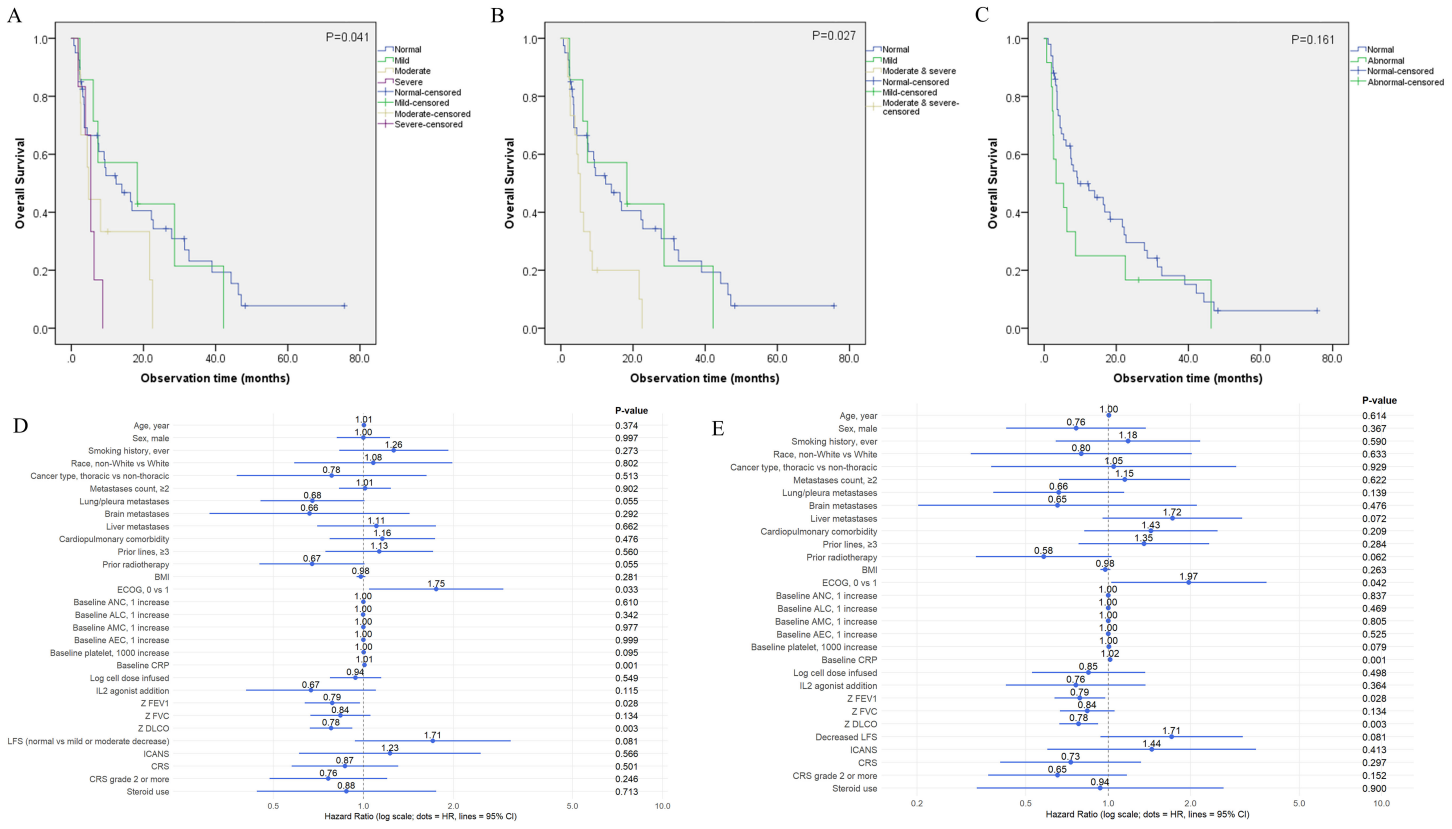
Overall survival according to DLCO and FEV_1_ Z-score categories (ABC). **(A)** Kaplan–Meier curves for overall survival stratified by DLCO Z-score categories (normal, mild, moderate, and severe impairment). **(B)** Kaplan–Meier curves for overall survival comparing normal, mild, and combined moderate/severe impairment groups based on DLCO Z-score. **(C)** Kaplan–Meier curves for overall survival comparing normal and abnormal groups based on FEV_1_ Z-score. **(D)** Forest plot of univariate Cox proportional hazards analyses for overall survival in the overall cohort. **(E)** Forest plot of univariate Cox proportional hazards analyses for overall survival in the pulmonary function test subgroup. DLCO, diffusing capacity of the lung for carbon monoxide; FEV_1_, forced expiratory volume in 1 second; OS, overall survival; PFT, pulmonary function test.

The median OS of the overall cohort was 8.6 months (95% CI, 6.8–10.4 months). **Figure 1** presents Kaplan–Meier curves stratified by zDLCO and zFEV1 category. The log-rank test across all categories showed a significant difference in survival (*P* = 0.041) (**Figure 1A**). When the moderate and severe categories were combined, the log-rank test remained significant (*P* = 0.028) (**Figure 1B**). When OS was compared between normal and abnormal zFEV1 groups, no significant difference was present (*P* = 0.161) (**Figure 1C**). **Figure 1D** shows univariate analysis of risk factors for OS, where ECOG performance status, baseline CRP, zFEV1, and zDLCO were statistically significant. In the multivariate model, 117 patients were included, and baseline CRP and ECOG were entered. Baseline CRP was significantly associated with OS (HR 1.009, 95% CI 1.005–1.013; *P* < 0.001) (**Table 2**).

**Table 2:** Multivariate analysis, OS in overall patients.

Variable	Univariate	Multi-Model	N (117)
	P-value	HR (95% CI)	P-value
Baseline CRP	<0.001	1.009 (1.005-1.013)	<0.001
ECOG	0.021	1.438 (0.846-2.442)	0.179

ECOG, Eastern Cooperative Oncology Group; CRP, C-reactive protein; HR, hazard ratio; CI, confidence interval.

The proportional hazards assumption was satisfied (global Schoenfeld residual test, χ² = 0.93, df = 2, P = 0.628).

In the PFT subgroup, univariate analyses (**Figure 1E**) showed that ECOG performance status, baseline CRP, zFEV1, and zDLCO were significantly associated with OS. In the multivariate model, 61 patients were included, and ECOG, baseline CRP and zDLCO were entered. Baseline CRP and zDLCO were significantly associated with OS (HR 1.016, 95% CI 1.009–1.022; *P* < 0.001 and HR 0.794, 95% CI 0.674-0.936; *P* = 0.006, respectively) (**Supplementary Table 2**).

### 3.4 PFS

**Figure 2:**
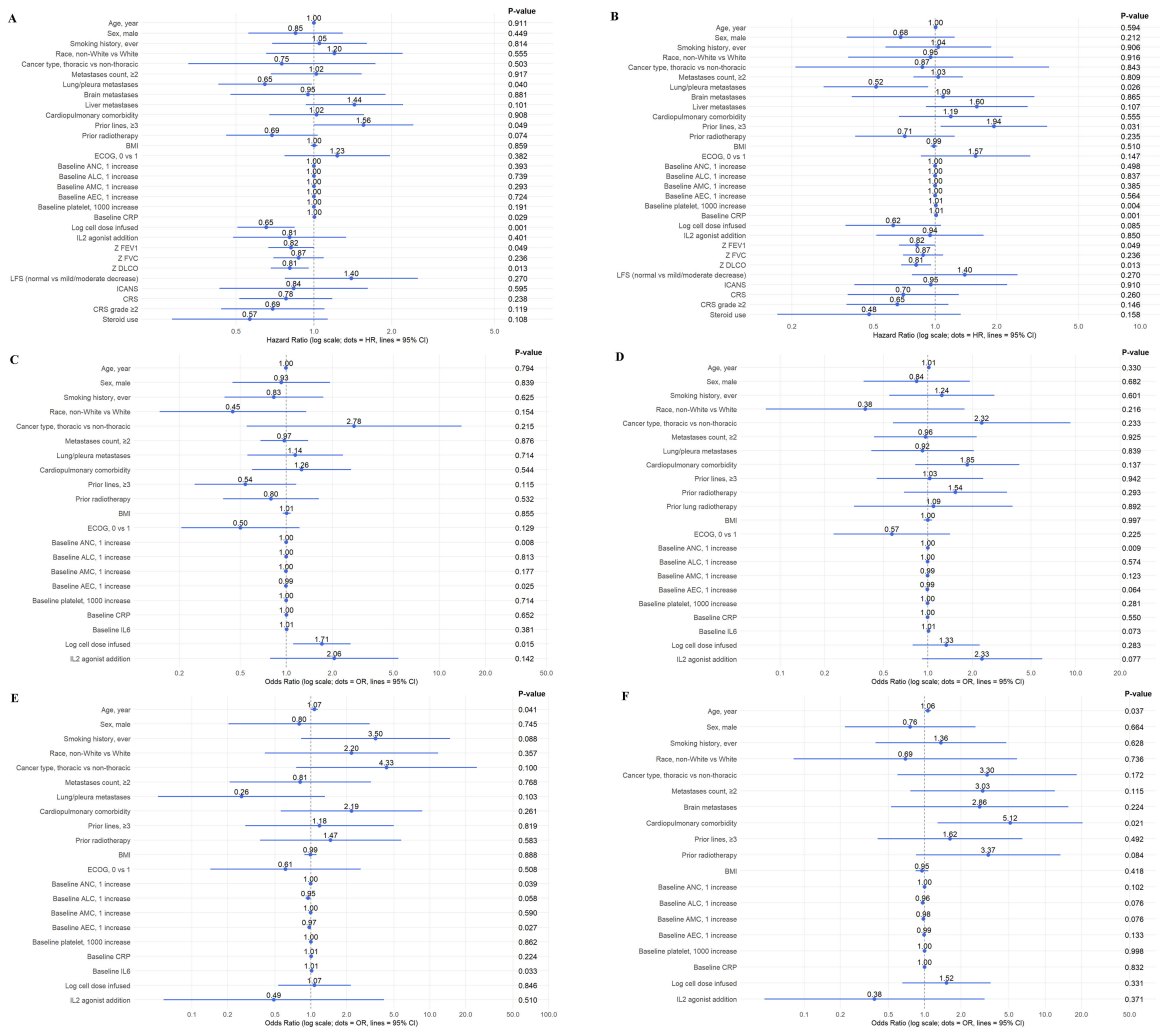
Univariate analyses of clinical predictors for progression-free survival and treatment-related toxicities in solid-tumor cellular therapy. **(A)** Forest plot of univariate Cox proportional hazards analyses for progression-free survival in overall patients. **(B)** Forest plot of univariate Cox proportional hazards analyses for progression-free survival in the pulmonary function test subgroup. **(C)** Forest plot of univariate logistic regression analyses for cytokine release syndrome of any grade. **(D)** Forest plot of univariate logistic regression analyses for cytokine release syndrome grade ≥2. **(E)** Forest plot of univariate logistic regression analyses for cytokine release syndrome grade ≥3. **(F)** Forest plot of univariate logistic regression analyses for immune effector cell–associated neurotoxicity syndrome. CRS, cytokine release syndrome; ICANS, immune effector cell–associated neurotoxicity syndrome; PFS, progression-free survival; PFT, pulmonary function test.

The median PFS of the overall cohort was 2.9 months (95% CI, 2.7–3.1 months). **Supplementary Figure 1** shows Kaplan–Meier curves for comparing PFS according to zDLCO categories and zFEV1. **Figure 2A** shows univariate analysis of risk factors for PFS. Lung or pleural metastases, a higher number of prior lines of therapy, and log-transformed cell dose showed significant associations. Additionally, zFEV1 and zDLCO showed significant associations with PFS. In the multivariate model, 75 patients were included, and in number of prior lines of therapy, log-transformed cell dose and baseline CRP were entered. Log-transformed cell dose showed a significant association with PFS (HR 0.636, 95% CI 0.476–0.850; P = 0.002) (**Table 3**).

**Table 3:** Multivariate analysis, PFS in overall patients.

Variable	Univariate	Multi-Model	N (75)
	P-value	HR (95% CI)	P-value
Prior lines, ≥3	0.040	1.003 (0.509-1.976)	0.994
Baseline CRP	0.029	1.004 (0.999-1.010)	0.155
Log cell dose infused	0.001	0.636 (0.476-0.850)	0.002

CI, confidence interval; CRP, C-reactive protein; HR, hazard ratio; HR, hazard ratio.

The proportional hazards assumption was satisfied (global Schoenfeld residual test, χ² = 1.03, df = 3, P = 0.795).

Analyses for association with PFS were performed for the subgroup with PFT results. **Figure 2B** shows univariate analysis of risk factors for PFS. Lung or pleural metastases, number of prior lines, baseline platelet count, baseline CRP, zFEV1, and zDLCO were significantly associated with PFS in univariate analyses. In the multivariate model, 59 patients were included, and prior treatment lines ≥ 3 (HR 1.894, 95% CI 1.029–3.486; *P* = 0.040) and baseline CRP (HR 1.012, 95% CI 1.006–1.017; *P* < 0.001) were associated with shorter PFS, while zDLCO (HR 0.826, 95% CI 0.696–0.979; *P* = 0.028) was significantly associated with longer PFS (**Supplementary Table 3**).

### 3.5 CRS and ICANS events

CRS was observed in 70 patients (57.4%) (**Table 4**). CRS severity was grade 1 in 37 patients (52.9%), grade 2 in 24 (34.3%), grade 3 in 7 (10.0%), and grade 4 in 2 (2.8%). In terms of respiratory support, 19 required low-flow oxygen, 7 required high-flow oxygen, 1 required noninvasive ventilation, and 1 required invasive mechanical ventilation. Six patients (4.9%) required admission to the intensive care unit for CRS management. Tocilizumab was administered to 31 patients (25.4%), and systemic corticosteroids to 12 patients (9.8%). ICANS developed in 11 patients (9.0%), most commonly of grade 1 (45.4%), followed by grades 3 (36.4%), 2 (9.1%), and 4 (9.1%).

**Table 4:** CRS and ICANS.

Parameter	N (%)
CRS	70 (57.4)
Grade	
1	37 (52.9)
2	24 (34.3)
3	7 (10.0)
4	2 (2.8)
Days between cell infusion and onset of CRS (median (range))	1 (0-22)
CRS management	
Highest oxygen requirement during CRS	
None	93 (76.2)
Nasal cannula ≤6	19 (15.6)
Nasal cannula >6	1 (0.8)
High flow	7 (5.7)
Non-invasive ventilation	1 (0.8)
Invasive ventilation	1 (0.8)
ICU admission due to CRS	6 (4.9)
Inotropic use	2 (1.6)
Steroid use	12 (9.8)
Tocilizumab	31 (25.4)
ICANS	11 (9.0)
Grade 1	5 (45.4)
Grade 2	1 (9.1)
Grade 3	4 (36.4)
Grade 4	1 (9.1)
ICU admission due to ICANS	3 (2.5)

CRS, cytokine release syndrome; ICANS, immune effector cell–associated neurotoxicity syndrome; ICU, intensive care unit.

### 3.6 Factors associated with CRS of any grade

**Figure 2C** presents the univariate analysis of factors associated with CRS of any grade. Baseline ANC and baseline AEC were associated with a lower risk of CRS. Additionally, infused cell dose was associated with a higher risk of CRS (OR 1.710, 95% CI, 1.111–2.632, *P* = 0.015). In the multivariate analysis, 122 patients were included, with infused cell dose, baseline ANC and AEC, and a number of prior lines entered as covariates. A lower baseline ANC was associated with an increased risk of CRS, with each 1000 cells/µL increase corresponding to lower odds (OR 0.594, 95% CI 0.376–0.939; P = 0.026), while infused cell dose showed a non-significant trend toward higher risk (OR = 1.469, 95% CI 0.943-2.290; *P* = 0.089) (**Table 5**).

**Table 5:** Multivariate models, CRS risk regardless of grade.

Variable	Univariate	Multivariate	N (122)
	P-value	OR (95% CI)	P-value
Log cell dose infused	0.045	1.469 (0.943-2.290)	0.089
Baseline ANC	0.008	0.594 (0.376-0.939)	0.026
Baseline AEC	0.025	0.023 (0.0002-2.284)	0.108
Prior lines, ≥3	0.115	0.975 (0.399-2.382)	0.955

ANC, absolute neutrophil count; AEC, absolute eosinophil count; CI, confidence interval; CRS, cytokine release syndrome; OR, odds ratio.

### 3.7 Factors associated with CRS grade 2 or more

**Figure 2D** shows the univariate analysis of factors associated with CRS grade ≥2. Baseline ANC showed significant association. In the multivariate logistic regression analysis for CRS grade ≥2, 122 patients were included, with baseline ANC and cardiopulmonary disease entered as covariates. A lower baseline ANC was significantly associated with CRS grade ≥2 (OR 0.470, 95% CI 0.252–0.877; *P* = 0.018).(**Supplementary Table 4**).

### 3.8 Factors associated with CRS grade 3 or more

**Figure 2E** presents the univariate analysis of associations with CRS grade ≥3. Age, baseline ANC, baseline AEC, and baseline IL-6 were significantly associated with CRS grade ≥3 (P = 0.041, 0.039, 0.027, and 0.033, respectively).

### 3.9 Factors associated with ICANS

**Figure 2F** shows univariate analysis for association with ICANS. Age and cardiopulmonary comorbidity were significantly associated with a higher risk of ICANS (P = 0.037 and 0.021, respectively).

### 3.10 Comparison between NK-cell and T-cell therapy groups

A total of 19 patients received NK cell therapy and 103 received T-cell therapy. Baseline characteristics, including age, sex, smoking history, and prior treatments, were comparable between the two groups. CRS of any grade occurred only in the T-cell group (68.0%; P<0.001), whereas no significant differences were observed in median PFS or OS (**Supplementary Table 5**).

## 4 Discussion

The present study analyzed predictors for survival outcomes, CRS, and ICANS in patients receiving cellular therapies for solid tumors. Higher baseline CRP levels and lower zDLCO were associated with shorter OS, while higher infused cell dose were associated with longer PFS. CRS occurred in over half of the patients, with lower baseline ANC correlating with an increased risk of CRS of any grade and CRS of grade 2 or higher.

We performed in-depth analyses of survival outcomes in both the overall cohort and the subgroup with available PFT results. zDLCO showed significant associations with OS and with PFS in the subgroup with available PFT results. While PFT parameters primarily reflect patients’ pulmonary function, they are also powerful predictors of all-cause mortality (24, 25), including mortality from cardiovascular causes (26, 27). Baseline pulmonary function may carry prognostic information relevant to outcomes after cellular therapy. Z-scores are race-agnostic and are adjusted for factors such as age, sex, and height (21), and they can be easily calculated using web-based calculators. Additionally, CRP levels which showed significant associations with PFS and OS, have been incorporated into several prognostic models for patients with hematologic malignancies undergoing CAR T-cell therapy (28, 29). However, the significance of zDLCO should be interpreted with caution, as its association with OS and PFS was observed only in the subgroup with available PFT results, which comprised approximately half of the cohort and may represent patients in better general condition.

CRS, a systemic inflammatory response driven by hyperactivation of immune effector cells and excessive circulating cytokine release, is one of the most common toxicities of cellular therapy (30, 31). In hematologic malignancies, CRS has been reported in majority of patients undergoing cellular therapy (3, 6, 7, 17). ICANS causes potentially life-threatening neurotoxicity in 20–70% of patients receiving CAR-T therapy (17, 32, 33). In our study, CRS occurred in 57.4% of patients, regardless of grade. Among those who developed CRS, about half experienced grade ≥2 events. While data on the severity of CRS after cell therapy in solid tumors are limited, the overall incidence of our study patients is lower than that reported in hematologic malignancies (2, 6–8, 34).

In analyses for association with CRS, decreased ANC was also significantly associated with severe CRS. The majority of patients underwent lymphodepletion prior to cell infusion. We assume that prolonged neutropenia resulting from pre-infusion therapy or the cell therapy itself may have contributed to a more severe clinical course (35). One possibility is that decreased neutrophil count and impaired neutrophil-mediated regulation of inflammation may result in dysregulated cytokine cascades, potentially contributing to a more severe CRS course (36). Infused cell dose showed a trend toward higher risk of CRS of any grade, although no association was observed with higher grade CRS. An association between high cell dose and CRS development has been demonstrated in multiple previous studies (6, 37, 38). In addition, the cell dose showed a significant association with PFS. Dose-dependent survival benefits have been reported in hematologic malignancies, including better OS with higher tisagenlecleucel doses in pediatric refractory/relapsed B-cell acute lymphoblastic leukemia (39). In the bb2121 trial, a CAR T-cell therapy targeting B-cell maturation antigen, multiple myeloma patients receiving higher doses (≥150 × 10^6^ CAR+ T cells) had longer PFS than those receiving lower doses (<150 × 10^6^ cells) (40). Prospective studies are needed to accurately validate the impact of cell dose on clinical outcomes and toxicity in solid tumors.

ICANS occurred in 9% of patients, with 3 of the 11 affected patients receiving ICU care. Although shown in univariate analyses, cardiopulmonary comorbidity showed an association with higher risk of ICANS. Our definition was based on treatment history for conditions associated with reduced cardiopulmonary function, and patients with lower physiologic reserve may have been more susceptible to ICANS. While this finding requires validation in larger population, this group of patients may require closer surveillance to enable early management.

A question we wanted to raise was whether modification in the current treatment practice of cellular therapy should be considered. In the study by Sdayoor et al. on B-cell lymphoma, pretreatment PFT results had limited utility in patients undergoing CD19 CAR T-cell therapy: FEV_1_, DLCO, and pulmonary comorbidity did not correlate with OS or PFS (41). On the other hand, a recent publication from our institution reported that higher LFS was associated with an increased risk of CRS. When adjusting for prior lines of therapy, both FEV_1_ and FVC were protective against ICANS risk, supporting that a combination of pre–CAR-T spirometry and corrected DLCO may help clinicians better assess the risk of toxicities (16). Furthermore, the value of PFT has been observed in patients undergoing HCT for hematologic malignancies (15). Based on our findings, pre-treatment pulmonary function may carry prognostic information relevant to outcomes after cellular therapy, which warrants prospective evaluation.

Why lung or pleural metastases were associated with a lower HR for progression remains unclear, and this finding should be interpreted with caution. The most likely explanation is that, in many cases, lung or pleural metastases were mutually exclusive with metastases at other sites, such as the brain and liver.

The strengths of this study include the use of a comprehensive database incorporating factors such as cell dose, detailed adjudication of outcomes, and, to our knowledge, the largest PFT dataset reported in solid tumors. Furthermore, all patients were assessed and managed for acute toxicities using a protocolized, multidisciplinary approach at MD Anderson Cancer Center.

The present study has several limitations. First, the retrospective design may result in bias due to unmeasured confounders. Second, the cohort was heterogeneous, spanning multiple tumor types, cell products, and varied IL-2 regimens. These groups were analyzed together because no single subtype was large enough for stratified analysis, and because CRS and ICANS are shared toxicity syndromes across adoptive cell therapies that can be examined as common endpoints irrespective of the construct. Our findings therefore require validation in larger, more homogeneous cohorts. Third, patients with severe cardiopulmonary disease, such as interstitial lung disease or heart failure, were excluded from treatment, which is consistent with clinical practice. Fourth, the relatively small sample size may limit statistical power, particularly with regards to findings regarding PFT parameters. Lastly, we analyzed the total infused cell dose using a log transformation due to the wide variation in dose ranges. However, heterogeneity in the timing and number of cell infusions may have influenced the results, and this effect requires further validation. To overcome abovementioned limitations, a prospective study that accounts for the mentioned limitations is needed to validate the findings.

Baseline parameters, such as CRP and pulmonary function, may help identify patients at higher risk of poor outcomes and toxicity during cellular therapy for solid tumors.

## Data Availability

The datasets presented in this article are not readily available because the limited sample size, combined with rare treatment types, means that combinations of clinical variables could potentially allow identification of individual patients. Requests to access the datasets should be directed to David S. Hong (dshong@mdanderson.org).
