# Supplementary material for: Clinical determinants of survival outcomes and acute toxicity after adoptive cellular therapy for solid tumors
Source: Front Immunol. 2026 Jul 29;17:1848516. doi: 10.3389/fimmu.2026.1848516 (PMC13461577; doi:10.3389/fimmu.2026.1848516)
Supplement: Supplementary file 1 [file DataSheet1.zip › Supplementary Figure.DOCX]

**Supplementary figure 1. Comparison of PFS according to DLCO and FEV1 Z-score categories**

**(A) Kaplan–Meier curves for PFS comparing normal, mild, and combined moderate + severe impairment groups based on DLCO Z-score. (B) Kaplan–Meier curves for PFS comparing normal and abnormal groups based on FEV1 Z-score. (C) Kaplan–Meier curves for PFS stratified by DLCO Z-score (normal, mild, moderate, and severe impairment).**

**Abbreviations: DLCO, diffusing capacity of the lung for carbon monoxide; PFS, progression-free survival**
