# Supplementary material for: Clinical determinants of survival outcomes and acute toxicity after adoptive cellular therapy for solid tumors
Source: Front Immunol. 2026 Jul 29;17:1848516. doi: 10.3389/fimmu.2026.1848516 (PMC13461577; doi:10.3389/fimmu.2026.1848516)
Supplement: Supplementary file 1 [file DataSheet1.zip › Supplementary Tables 1-5.DOCX]

**Supplementary Table 1. Comparison between PFT and non-PFT groups**

| Parameter | Non-PFT | PFT | P-value |
| --- | --- | --- | --- |
| Number | 59 | 63 |  |
| Demographics |  |  |  |
| Age (median (range)) years | 59 (20-84) | 57 (21-79) | 0.249 |
| Sex |  |  | 0.508 |
| Female | 34 (57.6) | 40 (63.5) |  |
| Male | 25 (42.4) | 23 (36.5) |  |
| Smoking history^a^ |  |  | 0.206 |
| Never smoker | 31 (55.4) | 42 (66.7) |  |
| Ever smoker | 25 (44.6) | 21 (33.3) |  |
| Metastases |  |  | 0.437 |
| 0 | 11 (18.6) | 8 (12.7) |  |
| 1 | 18 (30.5) | 25 (39.7) |  |
| 2 | 16 (27.1) | 21 (33.3) |  |
| 3 or more | 14 (23.7) | 9 (14.3) |  |
| Prior lines of treatment |  |  | 0.037 |
| 0 | 1 (1.7) | 5 (7.9) |  |
| 1 | 4 (6.8) | 12 (19.0) |  |
| 2 | 10 (16.9) | 13 (20.6) |  |
| 3 or more | 44 (74.6) | 33 (52.4) |  |
| ECOG |  |  | 0.031 |
| 0 | 9 (15.3) | 20 (31.7) |  |
| 1 | 50 (84.7) | 41 (65.1) |  |
| 2 or more | 0 (0) | 2 (3.2) |  |
| Cell therapy |  |  |  |
| IL-2 agonist addition to cell infusion | 6 (10.2) | 18 (28.6) | 0.011 |
| Toxicity |  |  |  |
| CRS of any grade | 26 (44.1) | 44 (69.8) | 0.004 |
| ICANS of any grade | 4 (6.8) | 7 (11.1) | 0.404 |
| Survival outcomes |  |  |  |
| Median PFS (95% CI, months) | 3.0 (2.3-3.7) | 2.9 (2.7-3.1) | 0.278 |
| Median OS (95% CI, months) | 8.4 (4.8-12.0) | 8.7 (5.6-11.8) | 0.132 |

aThree patients have missing data, and percentages were calculated among patients with available data. Abbreviations: CI, confidence interval; CRS, cytokine release syndrome; ECOG, Eastern Cooperative Oncology Group; ICANS, immune effector cell–associated neurotoxicity syndrome; IL-2, interleukin-2; OS, overall survival; PFS, progression-free survival; PFT, pulmonary function test.

**Supplementary Table 2.** **Multivariate analysis, OS in PFT subgroup**

|  | Univariate | Multivariate model | N (61) |
| --- | --- | --- | --- |
|  | P-value | HR (95% CI) | P-value |
| ECOG | 0.033 | 1.550 (0.774-3.103) | 0.216 |
| Baseline CRP | <0.001 | 1.016 (1.009-1.022) | <0.001 |
| zDLCO | 0.003 | 0.794 (0.674-0.936) | 0.006 |

Abbreviations: ECOG, Eastern Cooperative Oncology Group; zDLCO, z-score of diffusing capacity of the lung for carbon monoxide; CRP, C-reactive protein; HR, hazard ratio; CI, confidence interval

The proportional hazards assumption was satisfied (global Schoenfeld residual test, χ² = 0.37, df = 3, P = 0.947).

|  | Univariate | Multivariate model | N (59) |
| --- | --- | --- | --- |
|  | P-value | HR (95% CI) | P-value |
| Prior lines, ≥3 | 0.031 | 1.894 (1.029-3.486) | 0.040 |
| Baseline CRP | <0.001 | 1.012 (1.006-1.017) | <0.001 |
| zDLCO | 0.013 | 0.826 (0.696-0.979) | 0.028 |

**Supplementary Table 3. Multivariate analysis, PFS in PFT subgroup**

Abbreviations: CRP, C-reactive protein; HR, hazard ratio; CI, confidence interval; zDLCO, z-score of diffusing capacity of the lung for carbon monoxide

The proportional hazards assumption was satisfied (global Schoenfeld residual test, χ² = 1.41, df = 3, P = 0.702).

**Supplementary Table 4. Multivariate models, CRS grade 2 or more**

|  | Univariate | Multivariate | N (122) |
| --- | --- | --- | --- |
|  | P-value | OR (95% CI) | P-value |
| Baseline ANC | 0.009 | 0.470 (0.252-0.877) | 0.018 |
| Cardiopulmonary disease | 0.098 | 1.878 (0.805-4.377) | 0.145 |

Abbreviations: ANC, absolute neutrophil count; CI, confidence interval; CRS, cytokine release syndrome; OR, odds ratio

**Supplementary Table 5. Comparison between NK and T cell products**

| Parameter | NK | T cell | P-value |
| --- | --- | --- | --- |
| Number | 19 | 103 |  |
| Demographics |  |  |  |
| Age (median (range)) years | 53 (35-72) | 58 (20-84) | 0.337 |
| Sex |  |  | 0.789 |
| Male | 11 (57.9) | 63 (61.2) |  |
| Female | 8 (42.1) | 40 (38.8) |  |
| Smoking history^a^ |  |  | 0.395 |
| Never smoker | 10 (52.6) | 63 (63.0) |  |
| Ever smoker | 9 (47.4) | 37 (37.0) |  |
| Metastases |  |  | 0.744 |
| 0 | 2 (10.5) | 17 (16.5) |  |
| 1 | 8 (42.1) | 35 (34.0) |  |
| 2 | 5 (26.3) | 32 (31.1) |  |
| 3 or more | 4 (21.1) | 19 (18.4) |  |
| Prior lines of treatment |  |  | 0.454 |
| 0 | 0 (0) | 6 (5.8) |  |
| 1 | 1 (5.3) | 15 (14.6) |  |
| 2 | 4 (21.1) | 19 (18.4) |  |
| 3 or more | 14 (73.7) | 63 (61.2) |  |
| ECOG |  |  | 0.535 |
| 0 | 3 (15.8) | 26 (25.2) |  |
| 1 | 16 (84.2) | 75 (72.8) |  |
| 2 or more | 0 (0) | 2 (1.9) |  |
| Cell therapy |  |  |  |
| IL-2 agonist addition to cell infusion | 3 (15.8) | 21 (20.4) | 0.643 |
| Toxicity |  |  |  |
| CRS of any grade | 0 (0) | 70 (68.0) | <0.001 |
| ICANS of any grade | 0 (0) | 11 (10.7) | 0.135 |
| Survival outcomes |  |  |  |
| Median PFS (95% CI, months) | 2.6 (0.3-4.9) | 3.0 (2.8-3.2) | 0.978 |
| Median OS (95% CI, months) | 8.6 (7.3-9.9) | 8.4 (5.3-11.5) | 0.565 |

aThree patients have missing data, and percentages were calculated among patients with available data. Abbreviations: CI, confidence interval; CRS, cytokine release syndrome; ECOG, Eastern Cooperative Oncology Group; ICANS, immune effector cell–associated neurotoxicity syndrome; IL-2, interleukin-2; NK, natural killer; OS, overall survival; PFS, progression-free survival
